# Factor Influencing Knowledge and Attitude Toward Human Papillomavirus Vaccine Among Adolescent Girls in Gambella Town, Southwest Ethiopia: A Community‐Based Cross‐Sectional Study

**DOI:** 10.1002/hsr2.72277

**Published:** 2026-04-02

**Authors:** Lewegneh Wegayehu Tessema, Yazachew Moges Chekol, Mulat Belay Simegn, Sofonias Addis Fekadu, Chekol Asnake, Wondu Feyisa Balcha, Eyob Getachew Weldehawaryat

**Affiliations:** ^1^ Department of Midwifery Gambella Teachers Education and Health Science Gambella Ethiopia; ^2^ Faculty of Medicine, Dentistry and Health Science University of Melbourne Australia; ^3^ Department of Public Health Debre Markos University Debre Markos Ethiopia; ^4^ School of Optometry and Vision Science University of New South Wales Sydney Australia; ^5^ Department of Nursing Gambella Teachers Education and Health Science College Gambella Ethiopia; ^6^ Department of Midwifery Bahir Dar University Bahir Dar Ethiopia; ^7^ Department of Maternal and Child Health Gambella Hospital Gambella Ethiopia

**Keywords:** adolescent, attitude, HPV vaccine, knowledge

## Abstract

**Background and Aims:**

The human papillomavirus vaccine is a proven preventive measure against cervical cancer. Globally, HPV vaccine uptake remains low, and the problem is particularly serious in middle‐ and low‐income countries. There is limited evidence on knowledge and attitudes toward the HPV vaccine in Ethiopia. Therefore, this study aims to assess the knowledge and attitudes of adolescent girls regarding the HPV vaccine in Gambella town, Ethiopia.

**Methods:**

A community‐based cross‐sectional survey was conducted from April 2025 to June 2025 in Gambella town, Southwest, Ethiopia. The collected data were entered into EpiData version 4.6 and then exported to STATA statistical software version 17 for cleaning, coding, and formal analysis. Bivariable and multivariable analyses were used to examine the association. Odds ratio (OR), 95% confidence intervals (CI), and *p*‐values < 0.05 were deemed statistically significant.

**Results:**

In this study, 57.09% of the participants had good knowledge, and 55.6% showed favorable attitudes toward the HPV vaccine. Factors associated with knowledge of HPV vaccine include religion, wealth index, educational status, mother's occupational status, health care, mass media, peers, attending school, having a mobile phone, and mother's education. Factors independently associated with attitudes toward HPV vaccine included knowledge, attending school, mother education, family size, receiving information from school about HPV, mother education, and wealth index.

**Conclusion:**

In this study, 57.09% of participants demonstrated good knowledge and 55.6% expressed favorable attitudes toward the HPV vaccine. Enhancing school‐based education, mass media engagement, and family involvement is essential to improve HPV vaccine uptake and reduce cervical cancer burden among adolescent girls.

**List of Abbreviations**
AOR: adjusted odd ratio
CC: cervical cancer
CI: confidence interval
COR: crude odd ratio
FMOH: Federal Ministry of Health
HIV: human immuno deficiency
HPV: human papilloma virus
HPV: human papillomavirus vaccine
PCA: principal component analysis
VIF: variance inflation factor
WHO: World Health Organizations

## 1 Background

Human papillomavirus (HPV) is a common sexually transmitted infection of the reproductive system and a primary cause of cervical cancer [1]. In addition to cervical cancer, HPV infection is associated with other anogenital cancers, including vulvar, vaginal, anal, and penile cancers, as well as oropharyngeal cancers [2]. Cervical cancer is the fourth leading cause of death in women worldwide, with a proposed estimate of 604,000 people diagnosed with it in 2020; among them, 342,000 were dead [3]. HPV types 16 and 18 are responsible for approximately 70% of all cervical cancer cases globally [4]. Additionally, these HPV subtypes are implicated in the development of recurrent respiratory papillomatosis [5]. The vast majority of HPV infections are asymptomatic or sub‐clinical, a characteristic that has facilitated the rapid transmission and widespread dissemination of the virus within populations [6]. In sub‐Saharan Africa, cervical cancer is the leading cause of cancer‐related deaths among women, with an age‐standardized incidence rate of 131.1 per 100,000 in 2020 [3, 7]. Cervical cancer is the second most common malignancy in Ethiopia, affecting 20.9 million women, with an estimated 4648 new cases and 3235 deaths per year, according to Federal Ministry of Health (FMOH) [8].

HPV vaccination represents a highly effective strategy for preventing infection among women who have not previously been exposed to the virus [9]. According to the World Health Organization (WHO), the global target for 2030, 90% of girls to complete the full HPV vaccination series by age 15 and reducing cervical cancer incidence to fewer than four cases per 100,000 women but in the current study, actual coverage was below 30% [10, 11]. WHO recommends HPV vaccination primarily for girls aged 9–14 years, with females aged 15 years and older considered a secondary target population [12]. In 2018, Ethiopia first launched the HPV vaccine to all 14‐year‐old girls in health centers and schools [13]. National guidelines recommend two doses of the HPV vaccine administered 6 months apart, and the vaccine is now primarily delivered through a school‐based system [13]. Initiation of vaccination prior to age 17 was associated with a significant reduction in the risk of anal precancerous lesions and cervical cancer compared with no vaccination [14, 15]. Knowledge and attitudes have been identified as key determinants of future vaccine acceptance [6]. Raising awareness and fostering favorable attitudes toward the HPV vaccine are critical indicators for the effective prevention and control of cervical cancer [16, 17].

Factors influencing knowledge and attitudes toward the HPV vaccine include educational status, media exposure, parental education, wealth index, receipt of vaccination information, and mobile phone [16, 17, 18].

More than 80% of cervical cancer fatalities are diagnosed at a late stage due to a lack of understanding and attitudes about the disease, as well as insufficient preventative measures [19]. A lack of knowledge about the HPV vaccine contributes to the morbidity and mortality associated with HPV infection. Studies indicate that most adolescents are unaware of the vaccine, posing a significant barrier to vaccination [20]. Lack of knowledge and attitudes about HPV vaccine and infection make adolescents reluctant to uptake the HPV vaccine [21].

In Ethiopia, few study is conducted about HPV knowledge and attitude and most of them were facility‐based study facility‐based settings in which adolescents have better knowledge and attitude about health practice [22, 23]. Among the regions of Ethiopia, Gambella has the highest HIV prevalence [24]. Evidence revealed that the prevalence of HPV infection is higher among women living with HIV [25]. In Gambella, practices such as polygamy, levirate marriage (a practice where a man marries his deceased brother's widow), and a high prevalence of sexually transmitted infections are common. In the region, vaccination program is available in school, health facility and community; however, the population is unaware of vaccine and location of service [26], this might leads to higher HPV rate. Women living with HIV, including those receiving antiretroviral therapy, are 6 times more likely to develop cervical cancer than HIV‐negative women [27]. Additionally, in Ethiopia particularly in developing region like Gambella the vaccine is newly introduced and limited study conducted about knowledge and attitudes of HPV vaccine. Therefore, the aim of this study is to assess adolescent girls' knowledge and attitudes toward HPV vaccine in Gambella town.

## 2 Methods

### 2.1 Study Design and Study Period

A community‐based cross‐sectional survey was conducted from April 2025 to June 2025 in Gambella town, Southwest, Ethiopia. Gambella Region is one of the administrative regions of Ethiopia. It is bordered by the Oromia Region to the east, the South West Ethiopia Region to the south, and South Sudan to the west. The region covers an estimated area of 29,782.82 km^2^. Gambella town, the regional capital, is located approximately 768 km from Addis Ababa, the capital city of Ethiopia. Gambella town is administratively divided into five kebeles, which have an estimated total population of 65,396 in 2025. Among this population, 3040 of them were female adolescents.

Gambella town has two health centers, one general hospital, one primary hospital, and seven private clinics. In terms of educational structure, the town has nine elementary schools (four public and five private), five high schools (three public and two private), as well as three preparatory schools (two public and one private). The HPV vaccination program has been implemented in Gambella Town since 2018, which is delivered in both school‐based and community‐based strategies [13].

### 2.2 Population

#### 2.2.1 Source Population

All adolescent girls aged 14–19 years who were living in Gambella town.

#### 2.2.2 Study Population

All adolescent girls aged 14–19 years who were living in the selected kebeles of Gambella town during the data collection period.

### 2.3 Inclusion Criteria and Exclusion Criteria

All adolescent girls aged 14–19 years who were living in the selected kebeles of Gambella town during the data collection period were included in the study. Adolescent girls who were critically ill or unable to respond at the time of data collection were excluded.

### 2.4 Sample Size Determination and Sampling Procedure

The sample size was calculated using a single population proportion formula by taking knowledge of HPV vaccine (24.9%) from a previous study using the following statistical assumptions: margin of error of 4%, *Z*‐value of 1.96, 95% confidence level, 10% nonresponse, and design effect of 1.5, which gave a final sample size of 990. To select study participants, we use the cluster sampling technique, and about 825 participants are selected out of the total of 3040 female adolescents in Gambella. There are two clusters in each kebele of Gambella town. From each kebele, we selected one cluster randomly by using simple random sampling technique.

### 2.5 Variables of the Study

#### 2.5.1 Dependent Variable

Knowledge and attitude.

#### 2.5.2 Independent Variables

Independent variables were social‐demographic factors (age, mother education status, mother occupation, family wealth status, religion, attending school, mother educational level and father educational level, and student educational status), information related factor (health care provider, peer, school, mass media, having mobile phone, and parents), and health service‐related factors (distance to reach health facility).

### 2.6 Operational Definition

#### 2.6.1 Knowledge

Knowledge of the HPV vaccine was assessed using six structured questions, and knowledge scores were computed by giving 1 point to participants who correctly answered the questions and 0 to those who did not (for participants who answered “no”). A total knowledge score was calculated for each study participant. Respondents who scored above the mean were categorized as having good overall knowledge, whereas those who scored below the mean were categorized as having poor overall knowledge [16, 21].

#### 2.6.2 Attitude

A total of 11 items on a five‐point Likert scale were used to measure attitudes toward the HPV vaccine. Each item was scored from 1 (“strongly disagree”) to 5 (“strongly agree”). Then, attitude score was calculated for each participant ranging from 0 to 11. A composite attitudes variable was then generated based on the mean score, after checking the normality of the distribution. Participants who scored above the mean were categorized as having favorable attitudes, while those who scored below the mean were categorized as having unfavorable attitudes toward the HPV vaccine [16, 28].

Distance to reach health facility: ‐ This was measured from the report of respondents on the walking hours to the health facilities. This was coded 1 if mothers reported the walking hours to reach the nearby health facility to be ≥ 30 min, if less than 30 min is coded as 0 [29].

Family size: ‐ This is determined by average family size if greater than average family size code as 1 and less than or equal code as 0.

Wealth index: ‐ Was calculated using 28 data items on a household's ownership of selected assets, types of water access, and sanitation facilities using component principal analysis (PCA) divided into five equal parts as the poorest, poorer, middle, richer, and richest wealth index [30].

Exposure to mass media: Participants reporting engagement with at least one form of media (television, newspapers, or radio) at least once per week were classified as having media exposure [31].

### 2.7 Data Collection Tool and Procedures

The questionnaires were adapted by reviewing literatures [22, 23, 32, 33, 34, 35]. Data were collected using an interview‐administered structured questionnaire. The questionnaires were originally developed in English and then translated into Amharic. Later on, it was translated back to English to ensure its consistency. The data were collected using an interview guide and a pretested questionnaire after taking informed consent from parents and female adolescents about the purpose of the study. The data was collected by three female diploma midwives and one female BSc midwife supervisor. One day of training was given to the data collectors before data collection. This questionnaire was pretested on 50 participants of Bonga town with similar socioeconomic status, and then necessary amendments were done before actual data collection based on our pretest.

### 2.8 Data Processing and Analysis

The collected data were entered into EpiData version 4.6, then exported to STATA statistical software version 17 for analysis for cleaning, coding, and formal analysis. Descriptive statistics were described using frequencies, mean, and standard deviation and presented in tables and texts. Bivariable logistic regression analysis was carried out to identify factors associated with HPV knowledge and attitudes. Variables with *p*‐value ≤ 0.25 from the bivariable analysis were entered into a multivariable logistic regression model. Variables with a *p*‐value < 0.05 in the multivariable logistic regression model were considered statistically significant. Adjusted odds ratio with 95% CI was used to examine the strength and direction of association. Multicollinearity was checked using the Variance Inflation Factor (VIF), and there was no multicollinearity effect between independent variables. The income status of the participants was analyzed by using principal component analysis (PCA). All categorical variables and continuous variables were categorized as “0” and “1” based on the quantity of continuous variables. All statistical assumptions of factor analysis were checked, like Keiser–Meyer–Olkin (KMO) and Bartlett's test of sphericity. Any variable with less than a 0.5 communality value was removed from the analysis, and the analysis was done repeatedly until all variables were meeting the inclusion criteria for factor analysis. Next, all eligible factor scores were computed using the regression‐based method to generate one variable, wealth status. Following this, the final scores were ranked in five quantiles: first, second, third, fourth, and fifth. Finally, ranks were coded as richest, rich, middle, poorer, and poorest, respectively.

### 2.9 Ethics Approval and Consent to Participate

Ethical clearance was obtained from Gambella Teachers Education and Health Science College Ethical Review Committee. A permission letter was obtained from the Gambella health office for each kebele administrator office. Similarly, a support letter was obtained from Gambella town administration and given to kebele administration. After a detailed and brief explanation about the purpose and aim of the study, we received written informed consent from parent/guardian and assent from study participants during data collection time. We also informed that participants were voluntary and had the right to withdraw from the interview any time during data collection. All data obtained from study participants were kept confidential and assured for this study only. This study was conducted based on the Declaration of Helsinki.

## 3 Results

### 3.1 Socio‐Demographic Variable

Out of the total sample, 825 of study participants provided complete responses, resulting in a response rate of 83.3%. The mean age of adolescent girls was 16.6 with SD ± 1.49. Among the female adolescents, 759 (92%) of them were attending school. Most of the adolescents, 377 (45.7%) of them, were in primary school (Table 1).

**TABLE 1:** Socio‐demographic characteristics of female adolescents in Gambella town, Southwest Ethiopia, Ethiopia 2025.

Variables	Category	Frequency	Percent
Age	14	86	10.42
	15	123	14.91
	16	141	17.09
	17	208	25.21
	18	179	21.70
	19	88	10.67
Religion	Orthodox	311	37.70
	Muslim	119	14.42
	Protestant	267	32.36
	Catholic	128	15.52
Attending school	Yes	759	92
	No	66	8
Educational level	No formal education	12	1.45
	Primary (1–8)	377	45.70
	Secondary (9–12)	367	44.5
	Collage and above	69	8.36
Mother education level	No formal education	250	30.3
	Primary (1–8)	223	27.03
	Secondary (9–12)	136	16.48
	Collage and above	216	26.18
Mother occupation	House wife	351	42.55
	Farmer	28	3.39
	Merchant	189	15.39
	Private employee	127	15.39
	Governmental employee	130	15.76
Father education	No formal education	161	19.955
	Primary (1–8)	145	17.97
	Secondary (9–12)	125	15.97
	Collage and above	376	46.59
Father occupation	Farmer	136	23.79
	Merchant	192	16.85
	Private employee	176	21.81
	Governmental employee	288	35.69
	Other	15	1.86
Wealth status	Poorest	165	20
	Poorer	168	20.36
	Middle	162	19.34
	Richer	168	20.36
	Richest	162	19.64
Family size	≤ 5	521	63.15
	> 5	304	36.85
Distance to reach health facility	< 30 min	672	81.45
	≥ 30 min	153	18.55

### 3.2 Information Related Variables

From the total female adolescents, 221 (32.85%) of them were hearing about HPV vaccine from their health care providers. Near to half of adolescent girls, 369 (44.73%), were hearing about HPV vaccine from mass media. Almost all 737 (89.33%) of adolescent girls had mobile phones. From all study participants, only 124 (15.03%) of adolescents heard about HPV vaccine from school. Among the study participants, 69 (8.36%) of them heard about HPV vaccine from their friends.

### 3.3 Health Service‐Related Factor

Most of the respondents 672 (81.45%) lived near to health facilities; it takes less than 30 min to reach a health facility by walk, and 153 (18.55%) of them, it takes greater than 30 min by walk.

### 3.4 Knowledge of Adolescent Girls About HPV Vaccine

From all study participants, about 471 (57.09%) of them had good knowledge of HPV vaccine. Nearly three‐fourths of the study participants, 543 (65.82%), knew that HPV vaccination prevented the development of cervical cancer. More than half of the 500 adolescent girls (60.61%) knew the ideal recommended time to start HPV vaccine. More than half, 535 (64.85), of study participants knew HPV vaccination is currently offered freely to adolescent girls (Table 2).

**TABLE 2:** Knowledge of adolescent girls about HPV vaccine in Gambella town, Southwest Ethiopia, Ethiopia 2025.

Variables	Category	Frequency	Percent
HPV vaccine can prevent the development of cervical cancer	Yes	543	65.82
	No	282	34.18
HPV vaccination is currently offered freely to adolescent girls	Yes	535	64.85
	No	290	35.15
Ideal recommended time to start HPV vaccine	Age 9–14 years	500	60.61
	18–21 years	147	17.82
	At any age group	1147	17.87
	I don't know	27	3.27
	Others	4	0.48
HPV vaccine is allowed in all religion	Yes	607	73.58
	No	218	26.42
HPV vaccine is most effective when provided before sexual initiation	Yes	519	62.91
	No	306	37.09
Dose of HPV vaccine has recommended over 6 months schedule	One dose	273	33.13
	Two doses	351	42.60
	Three doses	74	8.98
	Four doses	17	2.06
	I don't know	109	13.23
Knowledge of adolescent girls about HPV vaccine	Good knowledge	471	57.09
	Poor knowledge	354	42.91

### 3.5 Attitude of Female Adolescents Toward HPV Vaccine

Of the study participants, 459 (55.6%) demonstrated favorable attitudes toward HPV vaccination. About one‐fifth of the respondents, 161 (19.52%), agree that they were susceptible to HPV infection any time, and more than one‐third, 331 (40.12%) and 169 (20.48%), disagree and strongly disagree that they were susceptible to HPV infection any time, respectively. More than half of 456 (55.27%) of respondents agree that health professional counseling affects their decision to receive HPV vaccination. Slightly more than half of the respondents, 470 (56.97%), agree that being vaccinated against HPV infection reduces exposure to HPV infection. Regarding side effects, 220 (26.67%) of the respondents were afraid of the side effects of the HPV vaccination, but 33.28% of them disagreed with the side effects of the HPV vaccination. Overall, 459 (55.64%) of respondents have a favorable attitude toward HPV vaccination, and 366 (44.36%) of them have unfavorable attitudes toward HPV vaccination, by using an average mean score of 3.43 (Table 3).

**TABLE 3:** Attitude of adolescent girls about HPV vaccine in Gambella town, Southwest Ethiopia, Ethiopia 2025.

Variable	1 = strongly disagree *N* (%)	2 = disagree *N* (%)	3 = neutral *N* (%)	4 = agree *N* (%)	5 = strongly agree *N* (%)
Susceptible to HPV infection any time	169 (20.48)	331 (40.12)	132 (16.0)	161 (19.52	31 (3.88)
Thinks vaccination helps to prevent HPV infection	34 (4.12)	101 (12.24)	177 (21.45)	427 (51.76)	86 (10.42)
It is difficult to find an HPV vaccine in Ethiopia	83 (10.06)	279 (33.8)	158 (19.15)	249 (30.2)	56 (6.79)
HPV vaccine is safe and effective	40 (4.85)	75 (9.09)	185 (22.42)	379 (45.94)	140 (17.7)
Afraid of the side effects of the HPV vaccination	67 (8.12)	279 (33.82)	193 (23.39)	220 (26.67)	66 (8)
Vaccinated for HPV reduce the risk of having HPV infection	22 (2.67)	58 (7.03)	102 (12.36)	470 (56.97)	173 (20.9)
Health care provider counseling you about HPV vaccination helps you to decide take the vaccine	28 (3.39)	50 (6.06)	88 (10.67)	456 (55.27)	203 (24.6)
Information on HPV vaccine helps you to decide whether you should be vaccinated against HPV	21 (2.55)	71 (8.61)	126 (15.27)	474 (57.45)	133 (16.1)
Use the vaccine if it is available at the clinic free to adolescents	55 (6.67)	174 (21.09)	81 (8.82)	309 (37.45)	206 (24.9)
HPV vaccine promotes risky sexual behaviors among teenagers?	14 (1.7)	111 (13.45)	200 (24.45)	351 (42.55)	149 (18.1)
HPV vaccine can cause infertility in the future	22 (2.67)	92 (11.15)	238 (28.85)	259 (31.39)	214 (25.9)

### 3.6 Factor Associated With Knowledge of HPV Vaccine Among Female Adolescents

In the bivariable logistic analysis, variables such as religion, wealth index, educational status, mother's occupational status, health care, mass media, peer, attending school, having a mobile phone, and mother's education were included. However, in multivariable analysis, mass media, peer, mother's occupation, and attending school, were positively associated while religion is negativity associated with knowledge of HPV vaccine.

Adolescents who received information about HPV from a peer had 2.15 greater odds of better knowledge compared to those who didn't receive information from peers (AOR = 2.2, 95% CI = 1.4–3.76). The odds of having better knowledge of the HPV vaccine were higher among farmers compared with housewives (AOR = 2.63; 95% CI: 1.09–6.33). The odds of HPV vaccine knowledge were higher among merchants compared with housewives (AOR = 1.54; 95% CI: 1.01–2.33). Private employee were 1.85 greater odds of better knowledge compared to housewives (AOR = 1.83, 95% CI = 1.13–2.95). Adolescents who attending school were 2.09 greater odds of having better knowledge about HPV vaccine compared with who didn't attend school (AOR = 2.09, 95% CI = 1.16–3.76). Catholic religion followers had significantly lower odds of being knowledgeable about the HPV vaccine compared with Orthodox religion followers (AOR = 0.51; 95% CI: 0.32–0.80) (Table 4).

**TABLE 4:** Bivariate and multivariable logistic regression of knowledge of HPV vaccine among adolescent girls in Gambella town, Southwest Ethiopia, Ethiopia 2025.

Variable	Knowledge of HPV vaccine		COR [95% CI]	AOR [95%CI]	*p*‐value
	Good knowledge	Poor knowledge			
Religion					
Orthodox	186	125	1	1	
Muslim	78	41	1.28 (0.82–1.99)	1.30 (0.82–2.09)	0.267
Protestant	152	115	0.88 (0.64–1.23)	0.90 (0.63–1.29)	0.595
Catholic	55	73	0.51 (0.33–0.76)	0.51 (0.32–80)**	0.004
Mother education					
No formal education	130	120	1	1	
Primary school (1–8 G)	127	96	1.22 (0.85–1.75)	0.90 (0.59–1.36)	0.625
Secondary school (9–12)	89	47	1.74 (1.13–2.69)	1.53 (0.95–2.46)	0.077
College and above	125	91	1.27 (0.88–1.82)	0.92 (0.58–1.47)	0.732
Educational status					
No formal education	2	10	1	1	
Primary education	219	158	6.3 (1.49–32.06)	3.54 (0.72–17.48)	0.120
Secondary	222	145	7.65 (1.65–35.44)	3.77 (0.76–18.69)	0.104
College and above	28	41	3.41 (0.694–16.78)	1.48 (0.28–7.82)	0.640
Mother occupation					
Housewife	174	177	1	1	
Farmer	18	10	1.83 (0.82–4.07)	2.63 (1.09–6.33)*	0.031
Merchant	118	71	1.69 (1.17–2.42)	1.54 (1.0–2.33)*	0.045
Private employee	83	44	1.91 (1.26–2.92)	1.82 (1.13–2.95)*	0.014
Government employee	78	52	1.53 (1.0–2.29)	1.42 (0.85–2.37)	0.179
Wealth index					
Poorest	83		1	1	
Poorer	87		1.10 (0.71–1.69)	0.94 (0.58–1.54)	0.825
Middle	94		1.29 (0.83–1.98)	0.87 (0.53–1.45)	0.603
Richer	101		1.58 (1.02–2.45)	1.14 (0.68–1.93)	0.604
Richest	106		1.77 (1.14–2.75)	1.08 (0.63–1.87)	0.758
Exposure to mass media					
Yes	225	231	2.05 (1.54–2.72)	2.30 (1.40– 3.767)**	0.001
No	246	123	1	1	
Peer					
Yes	47	22	1.67 (0.98–2.83)	2.15 (1.21–3.80)	0.009
No	424	332	1	1	
Attending school					
Yes	445	314	2.18 (1.30–3.64)	2.09 (1.16–3.76)*	0.014
No	26	40	1		
Health care					
Yes	181	90	1.83 (1.35–2.47)	1.18 (0.70–1.99)	0.529
No	290	264	1		
Having mobile phone					
Yes	39	49	1.78 (1.14–2.77)	1.56 (0.92–2.61)	0.096
No	432	305	1		

Abbreviations: AOR, adjusted odds ratio; COR, crude odds ratio.

* *p*‐value < 0.05

** *p*‐value < 0.01.

### 3.7 Factor Associated With Attitude of Adolescent Girls Toward HPV Vaccine

In the bivariable logistic analysis, variables such as knowledge, attending school, mother education, family size, receiving information from school about HPV, mother education, and wealth index were independently associated with attitudes toward the HPV vaccine. However, in the multivariable logistic analysis, wealth index, knowledge, and receiving information from school about HPV, were positively associated with attitude toward the HPV vaccine.

The odds of a favorable attitude toward HPV vaccination were higher among the middle economic status (AOR = 1.91; 95% CI (1.19–3.07) compared to poorest one. The odds of favorable attitude toward HPV vaccine higher among richer (AOR = 2.01; 95% CI: 1.24–3.24) compared with the poorest wealth status. The odds of a favorable attitude toward HPV vaccination were higher among participants of middle economic status compared with those of the poorest status (AOR = 2.04; 95% CI: 1.28–3.26). Adolescents who had better knowledge about HPV vaccine were 2.18 times (AOR = 2.18, 95% CI = (1.63–2.9)) more likely to have a better attitude toward HPV vaccine. Adolescents who received health information about the HPV vaccine from school had significantly higher odds of reporting a favorable attitude toward the vaccine compared with those who did not (AOR = 1.99; 95% CI: 1.29–3.05) (Table 5).

**TABLE 5:** Bivariate and multivariable logistic regression of attitude of HPV vaccine among adolescent girls in Gambella town, Southwest Ethiopia, Ethiopia 2025.

Variable	Attitude of HPV vaccine		COR [95% CI]	AOR [95%CI]	*p*‐value
	Favorable attitude	Unfavorable attitude			
Mother education					
No formal education	128	122	1	1	
Primary school (1–8 G)	86	86	1.52 (1.05–2.19)	1.32 (0.88–1.99)	0.183
Secondary school (9–12)	71	71	1.87 (0.57–1.32)	0.70 (0.44–1.11)	0.132
College and above	87	87	1.41 (0.98–2.04)	1.13 (0.71–1.76)	0.614
Mother occupation					
Housewife	179	172	1		
Farmer	13	15	0.83 (0.38–1.80	0.78 (0.34–1.79)	0.556
Merchant	119	70	1.63 (1.14–2.34)	1.29 (0.86–1.95)	0.210
Private employee	67	60	1.07 (0.72–1.61)	0.97 (0.61–1.54)	0.913
Government employee	81	49	1.59 (1.05–2.39)	1.34 (0.81–2.21)	0.248
Wealth index					
Poorest	66	99	1	1	
Poorer	75	75	1.8 (1.16–2.78)	1.57 (0.99–2.50)	
Middle	63	63	2.45 (1.58– 3.81)	1.91 (1.19–3.07)**	0.007
Richer	60	60	2.6 (1.66–4.05)	2.01 (1.24–3.24)**	0.004
Richest	69	69	2.09 (1.34–3.23	1.49 (0.92–2.430)	0.103
Attending school					
Yes	430	329	1.67 (1.00–2.76)	1.18 (0.68–2.06)	0.545
No	29	37	1		
Family size					
0	429	355	0.44 (0.22–0.89)	2.84 (1.34–5.99)**	0.006
1	30	11			
Receiving information about HPV vaccine from school					
Yes	87	37	2.08 (1.38–3.14)	2.06 (1.34–3.16)**	0.001
No	372	329			
Knowledge					
Good knowledge	303	168	2.29 (1.73–3.03)	2.27 (1.68–3.05)**	0.000
Poor knowledge	156	198	1		

Abbreviations: AOR, adjusted odds ratio; COR, crude odds ratio.

* *p*‐value < 0.05

** *p*‐value < 0.01.

## 4 Discussion

This study assessed adolescent girls' knowledge and attitudes toward HPV vaccine in Gambella town. The findings of this study revealed that 57.09% of adolescent girls in Gambella town demonstrated good knowledge of the HPV vaccine. This finding is in line with a study conducted in Debre Birhan 59.9% [36], Jimma town 52.7% [17]. The possible reason might be health literacy of adolescents and methodological similarity. However, this study is higher than the study conducted in Central Ethiopia 39.1% [28], Ambo 24.9% [32], Debre Tabor 46% [21], Addis Ababa 41% [37], and Bahir Dar 45.3% [22]. The possible reasons might be socio‐demographic differences, study population, study time, and the health literacy label of adolescents.

On the other hand, this finding is lower than the study conducted in Malaysia 80% [38], China 77.6% [39], Jordan 62% [40], and Zimbabwe 87.47% [41]. This discrepancy may be explained by sociocultural or study population differences. For instance, studies conducted in China, Malaysia, and Jordan were among medical students, who generally have better knowledge about the HPV vaccine due to the health information provided in their medical courses. Another possible explanation is that those studies were facility‐based, which may have led to overestimation since participants had greater opportunities to access health information about the HPV vaccine through their schools. In contrast, our study was conducted in the community, regardless of school status, which may account for the lower knowledge levels observed.

In this study, 55.6% of adolescent girls had a favorable attitude toward HPV vaccine. This study is in line with study conducted in Ambo town 55.6% [16], Addis Ababa 57% [37]. However, this study is higher than study conducted in Jimma 31.4% [17], Addis Ababa 36.5% [42], central Ethiopia 40.2% [28], and Bahir Dar 16% [22]. This discrepancy might occur due to socio‐cultural difference and study period and literacy level of adolescent.

Adolescents who had followed mass media had better knowledge about HPV vaccine. This may be attributed to the broader health information disseminated through media channels, which enhances awareness and understanding among those with media exposure [43]. Adolescents who attended school demonstrated greater knowledge of the HPV vaccine compared to those who did not. This difference may be attributed to the increased opportunities school‐going adolescents have to access health information through structured platforms, such as mini‐media programs and HIV clubs, which serve as important channels for health education and awareness [17]. A study conducted in China revealed that school‐based health education is a highly effective approach for enhancing adolescents' knowledge about HPV infection and the HPV vaccine within a short period of time [44]. Similarly, a study from Ambo reported that adolescent students who received information through school were twice as likely to have good knowledge about the HPV vaccine compared with their counterparts [16]. Adolescents' parents who were farmers had better knowledge of HPV vaccination compared to housewives. The possible justification might be farmers being able to move and exchange information in the community, but housewives are usually limited to the house. adolescents' parents who are merchants had better knowledge of HPV vaccine compared with housewives. The possible reason might be the merchant had a chance to exchange information about HPV vaccination in the marketplace. Adolescents whose parents were private employees are associated with knowledge of HPV vaccine compared with housewives. This study is supported by a study conducted in Utah [45]. The possible reason might be that women private employees had the opportunity to get information from their customers. Being Catholic religion follower reduces the use of the HPV vaccine by 49%. The possible reason might be some bishops might oppose HPV vaccination for school attendants [46]. So, this habit might reduce the knowledge of HPV vaccination.

The middle wealth status of their family is associated with the attitude toward HPV vaccine when compared to the poorest status. The richer status of their family is associated with the attitude toward HPV vaccine when compared to the poorest status. A study showed that parents with low economic status are not willing to vaccinate their daughters compared to their richer counterparts [47]. This is because families who are poor have low levels of exposure to mass media messages and low health literacy levels, which leads to low levels of attitudes toward HPV vaccine.

Adolescents who receive information about HPV vaccine from school have a better attitude toward HPV vaccine compared to those who do not receive it. This finding is supported by a study conducted in Malaysia [48]. Adolescents who had better knowledge of the HPV vaccine had favorable attitude toward HPV vaccine. This study is supported by the study conducted in Debre Tabor [23]. The possible justification might be that knowledge is a proximate factor for attitude, and adolescents who have better knowledge of HPV vaccine may have a better attitude toward HPV vaccine. A study revealed that students with a positive attitude toward the HPV vaccine increased their chances of knowing about the HPV vaccine by 1.46 times [22]. Family size below average is associated with attitude toward HPV vaccine. The possible reason might be that larger families had more competing demands on resources than smaller families; this leads to a low attitude toward HPV vaccine. The other possible reason might be social and cultural norms.

### 4.1 Strengths and Limitations

All studies conducted in Ethiopia were facility‐based, but this study was conducted at the community level, which is generalizable with a large sample size. However, this study is a cross‐sectional study, which lacks the ability to show the cause‐and‐effect relationship of dependent and independent variables. Since this study is primary data, it is subject to recall bias.

## 5 Conclusion

In this study, 57.09% of participants demonstrated good knowledge and 55.6% expressed favorable attitudes toward the HPV vaccine. Schools should serve as primary platforms for disseminating accurate information on the HPV vaccine, complemented by mass media campaigns and family engagement to reinforce awareness. Addressing socioeconomic disparities and cultural influences is critical to fostering positive attitudes and informed decision‐making among adolescent girls. Strengthening these pathways will be pivotal in enhancing HPV vaccine uptake and, ultimately, in reducing the burden of cervical cancer within this population.

## Author Contributions

**Lewegneh Wegayehu Tessema:** conceptualization, methodology, software, formal analysis, data curation, writing – review and editing, writing – original draft, validation. **Yazachew Moges Chekol:** conceptualization, software, data curation, validation, writing – original draft. **Mulat Belay Simegn:** conceptualization, methodology, software, validation. **Sofonias Addis Fekadu:** conceptualization, software, data curation, supervision. **Chekol Asnake:** conceptualization, methodology, data curation, writing – original draft. **Wondu Feyisa Balcha:** conceptualization, methodology, formal analysis, writing – original draft. **Eyob Getachew Weldehawaryat:** conceptualization, formal analysis, data curation, supervision.

## Funding

The authors have nothing to report.

## Disclosure

The lead author Lewegneh Wegayehu Tessema affirms that this manuscript is an honest, accurate, and transparent account of the study being reported; that no important aspects of the study have been omitted; and that any discrepancies from the study as planned (and, if relevant, registered) have been explained.

## Conflicts of Interest

The authors declare no conflicts of interest.

## Data Availability

The data that support the findings of this study are available from the corresponding author upon reasonable request.

## References

[1] WHO . Comprehensive Cervical Cancer Control. 2014, https://apps.who.int/iris/bitstream/handle/10665/144785/9789241548953_eng.pdf.

[2] CDC . HPV (Human Papillomavirus) Vaccine 2021, https://www.cdc.gov/vaccines/hcp/current-vis/downloads/hpv.pdf. 23156238

[3] H. Sung , J. Ferlay , R. L. Siegel , et al., “Global Cancer Statistics 2020: GLOBOCAN Estimates of Incidence and Mortality Worldwide for 36 Cancers in 185 Countries,” CA: A Cancer Journal for Clinicians 71, no. 3 (2021): 209–249. 33538338 10.3322/caac.21660

[4] WHO . Human Papillomavirus and Related Diseases Report 2021, https://hpvcentre.net/statistics/reports/XWX.pdf.

[5] J. Smahelova , E. Hamsikova , V. Ludvikova , et al., “Outcomes After Human Papillomavirus Vaccination in Patients With Recurrent Respiratory Papillomatosis: A Nonrandomized Clinical Trial,” JAMA Otolaryngology–Head & Neck Surgery 148, no. 7 (2022): 654–661. 35653138 10.1001/jamaoto.2022.1190 PMC9164118

[6] H. Trottier and E. L. Franco , “The Epidemiology of Genital Human Papillomavirus Infection,” Vaccine 24, no. Suppl 1 (2006): 1–15. 16406226 10.1016/j.vaccine.2005.09.054

[7] WHO . Cervical Cancer, 2014, https://www.who.int/news-room/fact-sheets/detail/cervical-cancer.

[8] FMOH . Guideline for Cervical Cancer Prevention and Control in Ethiopia, 2015.

[9] S. S. C. Chan , B. H. Yan Ng , W. K. Lo , T. H. Cheung , and T. K. Hung Chung , “Adolescent Girls' Attitudes on Human Papillomavirus Vaccination,” Journal of Pediatric and Adolescent Gynecology 22, no. 2 (2009): 85–90. 19345913 10.1016/j.jpag.2007.12.007

[10] WHO . Global Strategy to Accelerate the Elimination of Cervical Cancer as a Public Health Problem, 2020, https://www.who.int/publications/i/item/9789240014107.

[11] J. Han , L. Zhang , Y. Chen , et al., “Global HPV Vaccination Programs and Coverage Rates: A Systematic Review,” EClinicalMedicine 84 (2025): 103290. 40547442 10.1016/j.eclinm.2025.103290 PMC12179740

[12] WHO , Human Papillomavirus, 2019, https://www.who.int/docs/default-source/immunization/vpd_surveillance/vpd-surveillance-standards-publication/who-surveillancevaccinepreventable-08-humanpapillomavirus-r2.pdf?sfvrsn=5d7bc4ca_10%26download=true#:~:text=The%20World%20Health%20Organization%20(WHO,%2Dlike%20particles%20(VLPs).

[13] WHO , Ethiopia Launches Human Papillomavirus Vaccine for 14 Year Old Girls, 2018, https://www.afro.who.int/news/ethiopia-launches-human-papillomavirus-vaccine-14-year-old-girls.

[14] L. Baandrup , T. Maltesen , C. Dehlendorff , and S. K. Kjaer , “Human Papillomavirus Vaccination and Anal High‐Grade Precancerous Lesions and Cancer‐A Real‐World Effectiveness Study,” JNCI: Journal of the National Cancer Institute 116, no. 2 (2024): 283–287. 37718496 10.1093/jnci/djad189

[15] J. Lei , A. Ploner , K. M. Elfström , et al., “HPV Vaccination and the Risk of Invasive Cervical Cancer,” New England Journal of Medicine 383, no. 14 (2020): 1340–1348. 32997908 10.1056/NEJMoa1917338

[16] G. A. Bulto , E. E. Chaka , E. Y. Roga , B. T. Debelo , M. M. Erena , and T. L. Tefu , Knowledge and Attitude of Human Papillomavirus Vaccination and Associated Factors Among Adolescent School Girls in Ambo Town, Oromia Region, Ethiopia, 2021. 2021, https://pubmed.ncbi.nlm.nih.gov/35830389.

[17] T. Biyazin , A. Yilma , A. Yetwale , B. Fenta , and Y. J. H. V. Dagnaw , “Knowledge and Attitude About Human Papillomavirus Vaccine Among Female High School Students at Jimma Town, Ethiopia,” Human Vaccines & Immunotherapeutics 18, no. 1 (2022): 2036522, https://pubmed.ncbi.nlm.nih.gov/35236252/. 35236252 10.1080/21645515.2022.2036522 PMC9009896

[18] M. Beyen , G. A. Bulto , E. E. Chaka , et al., “Human Papillomavirus Vaccination Uptake and Its Associated Factors Among Adolescent School Girls in Ambo Town, Oromia Region, Ethiopia, 2020,” PLoS One 17, no. 7 (2022): e0271237. 35830389 10.1371/journal.pone.0271237 PMC9278730

[19] WHO , Comprehensive Cervical Cancer Control, 2014, https://apps.who.int/iris/bitstream/handle/10665/144785/9789241548953_eng.pdf.

[20] A. Y. Loke , M. L. Kwan , Y. T. Wong , and A. K. Y. Wong , “The Uptake of Human Papillomavirus Vaccination and Its Associated Factors Among Adolescents: A Systematic Review,” Journal of Primary Care & Community Health 8, no. 4 (2017): 349–362. 10.1177/2150131917742299 PMC5932744 29161946

[21] G. M. Aragaw , G. L. Aynalem , S. A. Abiy , et al., “Knowledge and Attitude of Parents Towards the Human Papillomavirus Vaccine for Their Daughters and Associated Factors in Debre Tabor Town, Northwest Ethiopia: A Community‐Based Cross‐Sectional Study,” BMJ Open 15, no. 10 (2025): e088550. 10.1136/bmjopen-2024-088550 PMC12506197 41047255

[22] E. A. Lakneh , E. A. Mersha , M. B. Asresie , and H. G. Belay , “Knowledge, Attitude, and Uptake of Human Papilloma Virus Vaccine and Associated Factors Among Female Preparatory School Students in Bahir Dar City, Amhara Region, Ethiopia,” PLoS One 17, no. 11 (2022): e0276465. 36409675 10.1371/journal.pone.0276465 PMC9678319

[23] G. N. Mihretie , T. M. Liyeh , A. D. Ayele , H. G. Belay , T. S. Yimer , and A. D. Miskr , “Knowledge and Willingness of Parents Towards Child Girl HPV Vaccination in Debre Tabor Town, Ethiopia: A Community‐Based Cross‐Sectional Study,” Reproductive Health 19, no. 1 (2022): 136, https://reproductive-health-journal.biomedcentral.com/counter/pdf/10.1186/s12978-022-01444-4.pdf. 35689288 10.1186/s12978-022-01444-4 PMC9188100

[24] HIV Prevention in Ethiopia National Road Map 2018–2020 . HIV Prevention in Ethiopia National Road Map 2018–2020, 2018, https://ethiopia.unfpa.org/sites/default/files/pub-pdf/HIV%20Prevention%20in%20Ethiopia%20National%20Road%20Map%202018%20-%202020%20FINAL_FINAL.pdf.

[25] “Human Papillomavirus and Cancer,” WHO, 2024, https://www.who.int/news-room/fact-sheets/detail/human-papilloma-virus-and-cancer.

[26] T. Gelibo , S. Lulseged , F. Eshetu , et al., “Spatial Distribution and Determinants of HIV Prevalence Among Adults in Urban Ethiopia: Findings From the Ethiopia Population‐Based HIV Impact Assessment Survey (2017–2018),” PLoS One 17, no. 7 (2022): e0271221. 35819961 10.1371/journal.pone.0271221 PMC9491827

[27] D. Stelzle , L. F. Tanaka , K. K. Lee , et al., “Estimates of the Global Burden of Cervical Cancer Associated With HIV,” Lancet Global Health 9, no. 2 (2021): e161–e169. 33212031 10.1016/S2214-109X(20)30459-9 PMC7815633

[28] H. Humnesa , M. Aboma , N. Dida , and M. Abebe , “Knowledge and Attitude Regarding Human Papillomavirus Vaccine and Its Associated Factors Among Parents of Daughters Age Between 9–14 Years in Central Ethiopia, 2021,” Journal of Public Health in Africa 13, no. 3 (2022): 16, https://publichealthinafrica.org/index.php/jphia/article/download/434/467. 10.4081/jphia.2022.2129 PMC9614691 36313923

[29] G. Fisseha , Y. Berhane , A. Worku , and W. Terefe , “Distance From Health Facility and Mothers' Perception of Quality Related to Skilled Delivery Service Utilization in Northern Ethiopia,” International Journal of Women's Health 9 (2017): 749–756. 10.2147/IJWH.S140366 PMC5633329 29042819

[30] T. Alene , A. Atnafu , Z. A. Mekonnen , and A. Minyihun , “Acceptance of Human Papillomavirus Vaccination and Associated Factors Among Parents of Daughters in Gondar Town, Northwest Ethiopia,” Cancer Management and Research 12 (2020): 8519–8526. 32982444 10.2147/CMAR.S275038 PMC7502398

[31] Central Statistical Agency (CSA) . Ethiopia Demographic and Health Survey. Addis Ababa, Ethiopia, and Rockville, Maryland, USA: CSA and ICF; 2016, https://dhsprogram.com/pubs/pdf/FR328/FR328.pdf.

[32] E. Yohannes , M. W. Beyen , G. A. Bulto , et al., “Knowledge and Attitude Toward Human Papillomavirus Vaccination and Associated Factors Among Adolescent School Girls in Ambo Town, Ethiopia, 2021: A Multicenter Cross‐Sectional Study,” Health Science Reports 6, no. 6 (2023): e1305. 37266064 10.1002/hsr2.1305 PMC10230424

[33] E. G. Woldehawaryat , A. B. Geremew , and D. B. Asmamaw , “Uptake of Human Papillomavirus Vaccination and Its Associated Factors Among Adolescents in Gambella Town, Southwest, Ethiopia: A Community‐Based Cross‐Sectional Study,” BMJ Open 13, no. 9 (2023): e068441. 10.1136/bmjopen-2022-068441 PMC10481830 37669848

[34] E. Y. Ukumo , F. G. Woldehawariat , S. A. Dessalegn , D. M. Minamo , and G. G. Ukke , “Assessment of Knowledge About Human Papillomavirus Vaccination Among Primary School Girls in Arba Minch Town, South Ethiopia, 2020 an Institution‐Based Cross‐Sectional Study,” Cancer Management and Research 14 (2022): 2205–2214, https://www.tandfonline.com/doi/pdf/10.2147/CMAR.S359413. 35880169 10.2147/CMAR.S359413 PMC9308045

[35] M. T. Sinshaw , S. Berhe , and S. G. Ayele , “Knowledge and Attitude Towards Human Papillomavirus Vaccine and Associated Factors Among Mothers Who Have Eligible Daughters in Debre Markos Town, Northwest Ethiopia,” Infection and Drug Resistance 15 (2022): 781–793, https://www.dovepress.com/article/download/73390. 35264861 10.2147/IDR.S352440 PMC8901188

[36] A. W. Tsige , K. D. Ayenew , and S. G. Ayele , “Assessment of Knowledge and Perceptions of Human Papillomavirus Vaccine and Its Determinants Among Women Who Have Eligible Daughters in Debre Berhan City, Ethiopia: A Cross‐Sectional Study,” Frontiers in Oncology 14 (2024): 1348288. 38562169 10.3389/fonc.2024.1348288 PMC10982310

[37] T. Regasa , E. G. Sendo , and J. T. Deressa , “Human Papillomavirus Knowledge, Perception, and Willingness to Receive Vaccination Among Female University Students in Addis Ababa University, Ethiopia, 2022: A Cross‐Sectional Study,” SAGE Open Nursing 9 (2023): 23779608231193554. 37576942 10.1177/23779608231193554 PMC10413898

[38] V. B. Badgujar , F. S. Ahmad Fadzil , H. K. Balbir Singh , F. Sami , S. Badgujar , and M. T. Ansari , “Knowledge, Understanding, Attitude, Perception and Views on HPV Infection and Vaccination Among Health Care Students and Professionals in Malaysia,” Human Vaccines & Immunotherapeutics 15, no. 1 (2019): 156–162, https://www.tandfonline.com/doi/full/10.1080/21645515.2018.1518843. 30199299 10.1080/21645515.2018.1518843 PMC6363156

[39] Y. Liu , N. Di , and X. Tao , “Knowledge, Practice and Attitude Towards HPV Vaccination Among College Students in Beijing, China,” Human Vaccines & Immunotherapeutics 16, no. 1 (2020): 116–123, https://www.tandfonline.com/doi/full/10.1080/21645515.2019.1638727. 31373874 10.1080/21645515.2019.1638727 PMC7012165

[40] M. Sallam , K. Al‐Mahzoum , H. Eid , et al., “Attitude Towards HPV Vaccination and the Intention to Get Vaccinated Among Female University Students in Health Schools in Jordan,” Vaccines 9, no. 12 (2021): 1432, https://mdpi-res.com/d_attachment/vaccines/vaccines-09-01432/article_deploy/vaccines-09-01432.pdf?version=1638535268. 34960177 10.3390/vaccines9121432 PMC8707789

[41] P. Zibako , N. Tsikai , S. Manyame , and T. G. Ginindza , “Knowledge, Attitude and Practice Towards Cervical Cancer Prevention Among Mothers of Girls Aged Between 9 and 14 Years: A Cross Sectional Survey in Zimbabwe,” BMC Women's Health 21, no. 1 (2021): 426, https://bmcwomenshealth.biomedcentral.com/counter/pdf/10.1186/s12905-021-01575-z.pdf. 34930221 10.1186/s12905-021-01575-z PMC8691087

[42] N. Dereje , A. Ashenafi , A. Abera , et al. Knowledge and Acceptance of HPV Vaccination and Its Associated Factors Among Parents of Daughters in Addis Ababa, Ethiopia: A Community‐Based Cross‐Sectional Study. 2021;16(1):1–7, https://pubmed.ncbi.nlm.nih.gov/34479576/. 10.1186/s13027-021-00399-8 PMC8418033 34479576

[43] B. J. Kelly , A. E. Leader , D. J. Mittermaier , R. C. Hornik , and J. N. Cappella , “The HPV Vaccine and the Media: How Has the Topic Been Covered and What Are the Effects on Knowledge About the Virus and Cervical Cancer?,” Patient Education and Counseling 77, no. 2 (2009): 308–313. 19395221 10.1016/j.pec.2009.03.018 PMC4250971

[44] X. Zhang , C. Liu , Z. Wang , et al., “Effect of a School‐Based Educational Intervention on HPV and HPV Vaccine Knowledge and Willingness to Be Vaccinated Among Chinese Adolescents: A Multi‐Center Intervention Follow‐Up Study,” Vaccine 38, no. 20 (2020): 3665–3670, https://pubmed.ncbi.nlm.nih.gov/32245644/. 32245644 10.1016/j.vaccine.2020.03.032

[45] D. Kepka , J. Bodson , D. Lai , et al., “Diverse Caregivers' HPV Vaccine‐Related Awareness and Knowledge,” Ethnicity & Health 26, no. 6 (2021): 811–826, https://pubmed.ncbi.nlm.nih.gov/30589389/. 30589389 10.1080/13557858.2018.1562052 PMC6597331

[46] The National Catholic Bioethics Center , Making Sense of Bioethics: Column 077: Vaccinating Our Children for Sexually Transmitted Diseases? 2011, https://www.ncbcenter.org/making-sense-of-bioethics-cms/column-077-vaccinating-our-children-for-sexually-transmitted-diseases.

[47] S. J. B. Hanley , E. Yoshioka , Y. Ito , et al., “Acceptance of and Attitudes Towards Human Papillomavirus Vaccination in Japanese Mothers of Adolescent Girls,” Vaccine 30, no. 39 (2012): 5740–5747. 22796375 10.1016/j.vaccine.2012.07.003

[48] M. Barnard , P. George , M. L. Perryman , and L. A. Wolff , Human Papillomavirus (HPV) Vaccine Knowledge, Attitudes, and Uptake in College Students: Implications From the Precaution Adoption Process Model. 2017;12(8):e0182266, https://pubmed.ncbi.nlm.nih.gov/28786994/. 10.1371/journal.pone.0182266 PMC5546631 28786994

